# Influence of age, gender, and race on nitric oxide release over acupuncture points-meridians

**DOI:** 10.1038/srep17547

**Published:** 2015-12-01

**Authors:** Sheng-Xing Ma, Paul C. Lee, Isabelle Jiang, Eva Ma, Jay S. Hu, Xi-Yan Li

**Affiliations:** 1 LA BioMed Research Institute at Harbor-UCLA Medical Center and Department of Obstetrics and Gynecology, David Geffen School of Medicine at UCLA and Harbor-UCLA Medical Center, Torrance, CA 90502

## Abstract

This study examined the influence of age, gender and race on nitric oxide (NO) release over acupuncture points, meridian without acupoint, and non-meridian regions of the Pericardium (PC) and Bladder (BL) meridian as well as aging on LU meridian in 61 healthy subjects. Biocapture tubes were attached to the skin surface, and total nitrite and nitrate was biocaptured and quantified using chemiluminescence. In elder ages compared to adults, NO levels over the ventral forearm were significantly decreased over LU on radial regions but not altered over PC on medial regions. Conversely, NO content was elevated over BL regions only in overweight/obesity of elder ages. NO levels over PC regions were marginally elevated in overweight/obese males compared to females but did not alter between races. These results suggest a selective reduction of NO release over LU meridian with aging, which is consistent with a progressive decline in lung function and increase in chronic respiratory disease in elder ages. Increased NO levels along the BL meridian in older obese subjects may reflect a modified NO level along somatic-bladder pathway for counteracting bladder dysfunctions with aging. Both of them support somatic-organ connections in the meridian system associated with potential pathophysiological changes with aging.

The Meridian System (Jingluo) is described by Traditional Chinese Medicine (TCM) as an essential pathway system, and the meridian system serves as the core theory behind various therapies such as acupuncture, acupressure, moxibustion, and Tai Chi/Qi Gong^1^,^2^. Major acupuncture points (acupoints) are located on the skin along 12 main meridian pathways, where vital energy “qi” is guided and believed to pass through a connection from the somatic meridian to the internal organs as a whole. Stimulation of acupoints has effects on the regulation of organ functions corresponding to related meridians. Anatomical studies have identified that the number of nerve fibers/trunks, blood vessels, hair follicles, and sweat glands are enhanced over acupoints compared to their adjacent control areas^3^,^4^. Despite numerous research endeavors with modern science on the meridian system, only the phenomena of meridians has been confirmed^1^,^5^,^6^,^7^, and the structure, biomolecules, and functions of the pathway, especially the relationship between the somatic meridian and internal organs, still remain unclear.

Nitric oxide (NO) plays important physiological roles in neurotransmissions and in the local control of microcirculation, immunity and wound healing on skin^8^,^9^. The skin represents a unique non-systemic site for the cutaneous measurement of NO metabolites and other biomarkers^10^,^11^,^12^,^13^. Weighing 10–12 kg on average, the skin is the largest reservoir for NO derivatives and donors, comprised of S-nitrosothiols (RSNOs), N-nitroso compounds, nitrite, and nitrate^14^,^15^. In skin, nitrite and RSNO are 25 and 360 times higher, respectively, compared to the plasma of healthy volunteers^12^. Consistently, there is a strong disproportionate variation of NO-related products among sweat, epidermis, plasma, and superficial vascular dermis^15^,^16^. In human skin cells, NO can be produced enzymatically by three nitric oxide synthases (eNOS, nNOS, iNOS) and other enzymes^14^, or non-enzymatically through the nitrate-nitrite-nitric oxide pathway^12^,^13^,^14^,^15^. NO, with a half-life of a few seconds, is spontaneously oxidized and stored as NO derivatives and donors over time through various mechanisms^14^,^15^.

Our previous studies have demonstrated that NOS protein levels and NO contents are consistently higher in skin regions containing acupoints/meridian lines compared to control tissues in rats^16^,^17^. Our recent studies demonstrated, using a biocapture device, that total nitrite and nitrate (NO_x_^−^) are quantifiable over skin regions of the forearm and leg in humans, and NO contents are higher over acupoints compared to meridian lines without acupoint (MWOP) and non-meridian control region (NMCR)^18^. The investigation of NO bioavailability over the skin surface is validated in other studies^19^, and is a potential physiological indicator for therapeutic manipulation of the skin microvasculature and in pathophysiology^11^,^17^. In this study, the influence of age, gender, and race on topographical distribution of NO were examined and analyzed over the skin surface of acupoints, MWOP, and NMCR along the Pericardium (PC) meridian regions of the ventral forearm and the Bladder (BL) meridian regions of the calf, as well as aging over Lung (LU) meridian regions, in human participants. Here we report an unexpected finding that NO levels biocaptured over the LU and BL meridian region are selectively modified by aging but over PC regions are elevated in overweight/obese males compared to females.

## Results

### Characteristics of Participants and Biocapture Regions

The study included 61 consecutive human subjects with 30 normal weight healthy volunteers (BMI < 25 kg/m^2^) and 31 overweight/obese subjects (BMI ≥ 25 kg/m^2^). The characteristics of the participants are detailed in Table 1. Body weight was significantly higher in overweight/obese subjects compared to normal weight subjects. The total population’s BMI was 21.3 ± 0.4 kg/m^2^ in normal weight healthy volunteers and 30.0 ± 0.9 kg/m^2^ in overweight/obese subjects. In the adult group, there was a significant increase in systolic and diastolic arterial blood pressure in overweight/obese subjects compared to normal weight subjects (P < 0.01). In normal weight subjects, there was only a significant increase in systolic arterial blood pressure in the elder age group compared to the younger age group (P < 0.05). Compared to overweight/obese subjects, normal weight subjects were about 3 years younger, and more Asian subjects participated in this study.

**Figure 1:**
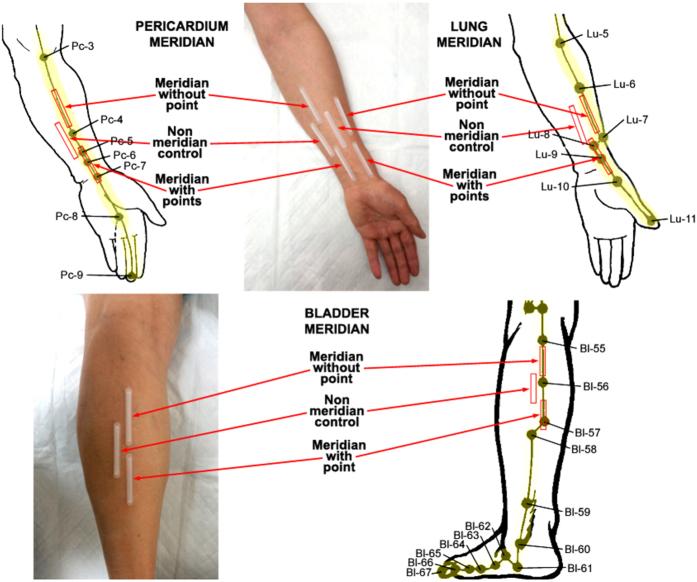
Representation of a biocapture device with a semi-cylindrical molded tube taped to the skin surface over meridians. The pericardium (PC), lung (LU), and bladder (BL) meridian lines and related acupuncture points are illustrated on the top left, top right, and bottom right panels. The region from PC 5 to PC 7 is defined as acupoint (3 acupoints), the distance between PC 3 and PC 4 is defined as meridian line without acupoint (MWOP), and non-meridian control region close to PC meridian (NMCR). The region overlapping LU 8 and LU 9 is defined as acupoint (2 acupoints), the distance between LU 6 and LU 7 represents MWOP, and NMCR as non-meridian area adjacent to the meridian. The region over BL 57 is defined as acupoint (1 acupoint), the distance between BL 55 and BL 56 represents MWOP, and NMCR as non-meridian area adjacent to the meridian. The device was adhered to the skin surface using a custom double-sided adhesive, and NO scavenging solution (100 μM PTIO) was injected into the tubing over the skin surface for 20 min in order to directly absorb NO. The PC, LU, and BL meridian lines and related acupuncture points are reproduced from J. A. Johnson, Chinese Medical Qigong Therapy: A Comprehensive Clinical Text, 2000.

Figure 1 is a representative example of the defined PC, BL, and LU acupoints, MWOP, and NMCR. A biocapture device with a semi-cylindrical molded tube was adhered to the skin surface (top central and bottom left panels) over the PC regions (top left panel) and LU regions (top right panel), and BL regions (bottom right panel).

### Influence of Age on NO Releases over LU, PC, and BL Regions

**Figure 2:**
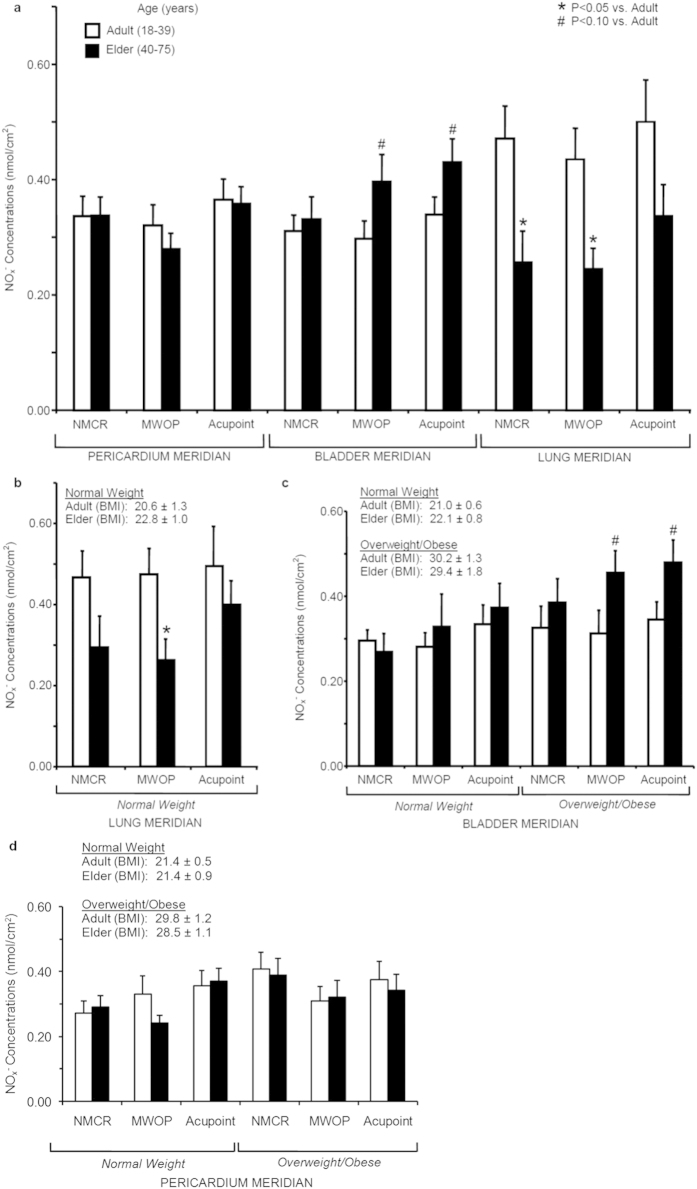
Quantification of nitric oxide (NO) metabolites over skin acupoints/meridians in elder subjects compared to adults. (**a**) Concentrations of total nitrite plus nitrate (NO_x_^−^) wee compared in adults vs. elder subjects over the skin regions along the Pericardium meridian (PC, n = 14 and 10), Bladder meridian (BL, n = 12 and 7), and Lung meridian (LU, n = 14 and 8). (**b**) NO_x_^−^ concentrations over LU regions were compared in normal weight adult vs. elder subjects (n = 10 and 16). (**c**) NO_x_^−^ concentrations over BL regions were compared between adult and elder subjects in normal weight (n = 12 and 6) and in overweight/obesity (n = 12 and 7). (**d**) NO_x_^−^ concentrations over PC regions were compared between adult and elder subjects in normal weight (n = 16 and 8) and in overweight/obesity (n = 12 and 10). NO_x_^−^ concentrations (nmol/cm^2^) were obtained over acupoints, meridian lines without acupoint (MWOP), and non-meridian control region (NMCR). Each bar represents the mean values and vertical bars represent S.E.M. *p < 0.05, compared to adult; ^#^p < 0.10, compared to adult.

NO_x_^−^ concentrations over LU, PC, and BL regions were examined in elder (age: 40–75 years) compared to adult (age: 18–39 years) groups in 61 healthy volunteers (Fig. 2a). ANOVA analysis of NO_x_^−^ concentrations biocaptured over LU regions in two age groups suggest a significant reduction of NO metabolites in the elder ages compared to adults (F_1,12_ = 7.5, P = 0.02). In contrast to LU regions, there were no detectable changes in NO content over PC regions in the elder ages compared to adults. NO levels over LU MWOP and NMCR were significantly decreased in elder compared to adult subjects (P < 0.01). NO content over LU acupoints tended to be reduced in elder compared to adult subjects, but fell short of statistical significance (P = 0.12).

In order to avoid the influence of overweight/obesity on modification of NO levels over skin, NO_x_^−^ concentrations over LU acupoints, MWOP, and NMCR between two age groups were compared in normal weight subjects alone, as shown in Fig. 2b. A two-way ANOVA revealed marginal differences in NO_x_^−^ concentrations between two age groups in normal weight subjects over LU regions (F_1,8_ = 2.0, P = 0.12). NO_x_^−^ concentrations in normal weight subjects were significantly reduced over MWOP and tended to be lower over LU NMCR in the elder subjects compared to the adult group (P = 0.04, P = 0.13, respectively). However, NO_x_^−^ level over LU acupoints was not significantly influenced by age in normal weight subjects.

There were marginal changes in NO content over BL regions in the elder ages compared to adults in overweight/obese subjects. As shown in Fig. 2c, right panel, NO_x_^−^ concentrations tended to be increased over BL regions in the elder age group compared to the adult group in overweight/obese subjects; however, there were no detectable changes in NO_x_^−^ concentrations over BL regions in elder subjects compared to adults in normal weight subjects (Fig. 2c, left panel) as well as over PC regions in elder subjects compared to adults in normal weight and overweight/obese subjects, respectively (Fig. 2d). ANOVA analysis of NO_x_^−^ concentrations over BL regions revealed no significant differences of NO metabolites in elder subjects compared to adults in normal weight subjects, and no significant differences of NO metabolites over PC regions in elder subjects compared to adults in normal weight and overweight/obese subjects. However, marginal increases in NO_x_^−^ concentration in the elder age group compared to adult group were detected in overweight/obese subjects over BL regions (F_1,17_ = 3.3, P = 0.09). NO_x_^−^ levels tended to be increased over BL acupoints and MWOP in the elder age group compared to adult group in overweight/obese subjects (P = 0.06, P = 0.07, respectively). In normal weight compared to overweight/obese subjects, NO_x_^−^ content over BL regions was not altered in adult group but was marginally increased in the elder age group (F_1,11_ = 4.4, P = 0.06).

### Influence of Gender on NO Releases over PC and BL Regions

**Figure 3:**
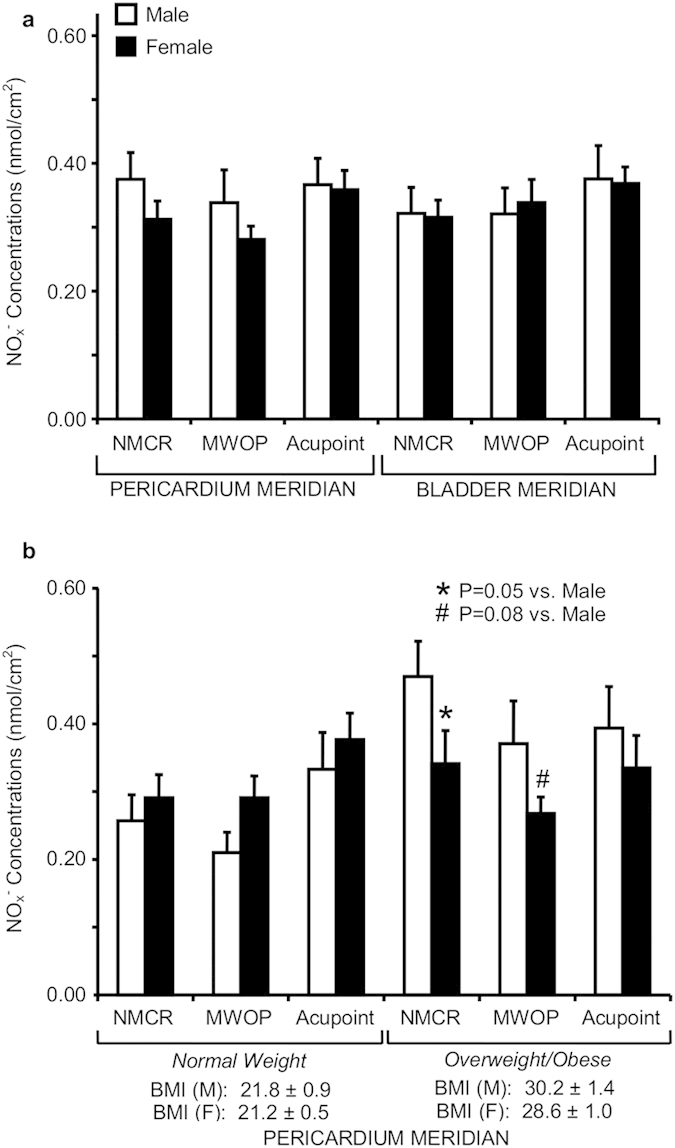
Quantification of nitric oxide (NO) metabolites over skin acupoints/meridians in male compared to female subjects. (**a**) Concentrations of total nitrite plus nitrate (NO_x_^−^) were compared between male and female subjects over the regions along the Pericardium (PC) meridian (n = 18 and 28) and Bladder (BL) meridian (n = 14 and 23). (**b**) NO_x_^−^ concentrations over PC regions between male and female subjects were compared in normal weight (n = 8 and 16) and overweight/obesity (n = 10 and 12). NO_x_^−^ concentrations (nmol/cm^2^) were obtained over acupoints, meridian lines without acupoint (MWOP), and non-meridian control region (NMCR). Each bar represents the mean values and vertical bars represent S.E.M. *p = 0.05, compared to male; ^#^p = 0.08, compared to male.

To determine the influence of gender on NO releases, NO_x_^−^ concentrations over PC and BL regions were compared between male and female subjects in 55 healthy volunteers, as shown in Fig. 3a. A two-way ANOVA revealed that there is no significant differences in NO_x_^−^ concentrations in males compared to females over PC and BL regions in normal weight plus overweight/obese subjects, but there is a marginally significant interaction between gender and BMI on NO_x_^−^ concentrations over PC regions only (F_1,41_ = 3.9, P = 0.06). In overweight/obese males, NO_x_^−^ concentration was marginally increased over PC regions compared to overweight/obese females (F_1,20_ = 2.6, P = 0.12), as shown in Fig. 3b. NO_x_^−^ levels over PC MWOP and NMCR in overweight/obese subjects were marginally and significantly elevated in males compared to females (P = 0.08, P = 0.05, respectively). NO_x_^−^ concentrations (nmol/cm^2^ Mean ± SE) over PC NMCR, MWOP, and acupoints in adult females of normal weight subgroup (BMI: 21.1 ± 0.66, n = 10) were 0.29 ± 0.05, 0.32 ± 0.05, and 0.34 ± 0.05, which are similar with 0.31 ± 0.06, 0.27 ± 0.04, and 0.33 ± 0.06 in adult female of overweight/obese subjects (BMI: 29.5 ± 1.43, n = 7). In adult males of normal weight versus overweight/obesity subgroups, the values of NO concentrations over PC acupoints, MWOP, and NMCR are higher in overweight/obesity subgroup than those in normal weight subgroup, but fell short of statistical significance (n = 4 in each subgroup). However, there were no detectable changes in NO contents over BL regions in males compared to females both in normal weight and overweight/obese groups.

### Influence of Race on NO Releases over PC and BL Regions

**Figure 4:**
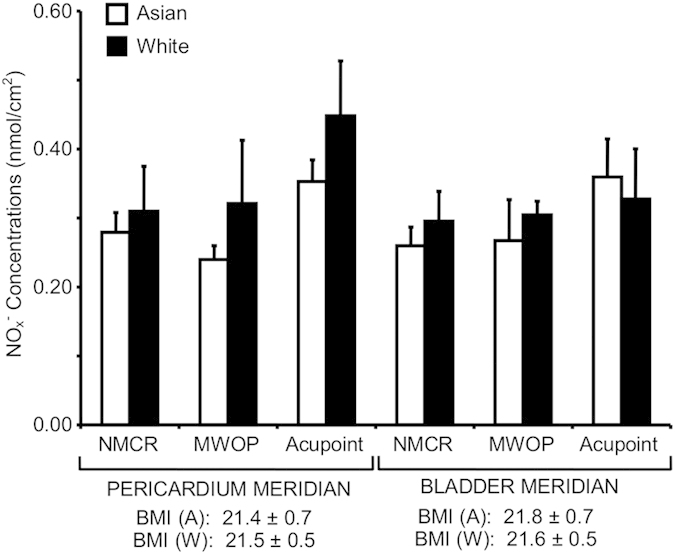
Quantification of nitric oxide (NO) metabolites over skin acupoints/meridians in Asian compared to white subjects. Concentrations of total nitrite plus nitrate (NO_x_^−^) were compared between Asian and white normal weight subjects over the regions along the Pericardium (PC) meridian (n = 16 and 6) and Bladder (BL) meridian (n = 9 and 5). NO_x_^−^ concentrations (nmol/cm^2^) were obtained over acupoints, meridian lines without acupoint (MWOP), and non-meridian control region (NMCR). Each bar represents the mean values and vertical bars represent S.E.M.

NO_x_^−^ concentrations were examined in six races along the skin surface of PC and BL regions in 50 healthy volunteers, as shown in Table 1. However, only Asian and white races have enough sample number for a comparison. Figure 4 shows NO_x_^−^ concentrations over PC and BL acupoints, MWOP, and NMCR in Asian races compared to white in normal weight subjects. ANOVA analysis of NO_x_^−^ concentrations over PC and BL regions in Asian compared to white subjects suggest no significant changes in NO release between the races.

## Discussion

The purpose of this study was to examine the topographical distribution of NO_x_^−^ biocaptured over the skin surface of acupoints, MWOP, and NMCR along PC, BL, and LU meridians. The influences of age, gender, and race on NO releases over PC, and BL meridian regions, as well as aging over LU meridian region, were compared in humans. The major new findings of these studies are: (1) NO_x_^−^ levels were selectively lower over LU regions in older subjects but unaltered over PC regions; (2) NO_x_^−^ levels over BL regions tended to be increased in older overweight/obese subjects; (3) NO_x_^−^ levels over PC regions in overweight/obese subjects tended to be elevated in males compared to females; and (4) NO release was not significantly different over PC and BL regions between white and Asian subjects. This is a post-hoc analysis of experimental data generated from the studies with double-blinding of both participants and researchers. The unexpected findings show that NO release is decreased in older ages over LU regions and the difference even occurs in normal weight elder compared to adult subjects. Interestingly, such kind of decreases with aging only occur along LU regions located on the radial side of the ventral forearm but are not changed over PC regions between adults vs. elder subjects in normal weight and overweight/obese subjects, the medial side of the same ventral forearm close to the LU region. NO contents biocaptured over the BL meridian tended to be higher in elder overweight/obese subjects compared to adults but was not altered in normal weight subjects. Moreover, enhanced NO over PC regions in overweight/obese males compared to females suggests gender differences on NO release over selective PC regions depending on body weight. This is the first evidence demonstrating that a selective reduction of NO release exists over LU regions with aging, which is independent to body weight, and body weight-dependent elevations of NO release occur over BL regions in older subjects and over PC regions in males. These findings suggest that topographical modifications of NO synthesis/release over LU, BL, and PC regions associate with age, gender, and obesity, which may be involved in potential pathophysiological changes in meridians and their corresponding organs.

The sources and mechanisms of modification in NO synthesis/release over skin regions associated with aging, gender, and body weight are still unknown. The present results show that NO levels are only reduced in LU regions, not altered over PC regions but enhanced in BL regions. In order to avoid the influence of overweight/obesity on modification of NO levels over skin, the NO levels over LU regions are compared between adult and elder subgroups only in normal weight subjects. NO levels over LU regions are less in elder than those in adult of normal weight subjects. The findings presented suggest that NO level is predominantly reduced from the skin surface of LU regions with aging, which is independent to body weight. However, a greater age-based change on the LU acupoint region was not observed, which involve at least two sources of NO, L-arginine-derived NO synthesis and non-enzymatic production of bacteria^12^,^13^,^14^,^15^,^16^,^18^, that contribute to the alteration of NO levels over skin regions. Assuming that a higher level of NO over acupoints is mainly from non-enzymatic NO production while reduction of NO level over Lung meridian region mainly results from impaired L-arginine-derived NO synthesis with aging, we speculate that a significant NO level difference in the meridian line without acupoint region and non-meridian control area can occur since such decreased L-arginine-derived NO over LU acupoints in elder subjects is blunted by higher level of non-enzymatic NO on the acupoints. Based on TCM, the Lung Meridian (LU) is a functional pathway between the radial side of the ventral forearm to the internal lung organ, and the lung is in charge of vital qi or life energy, and qi deficiency in the lung is associated with aging^2^. The present results from aging on the reduction of NO level over LU meridian region may reflect a somatic-lung organ connection in TCM, describing an association between the LU meridian and qi deficiency in the lung organ. It is well documented that age-related changes in the lungs result in a progressive decline in lung function, persistent low-grade inflammation in the lower respiratory tract, and reduction of anti-oxidants in the epithelial lining fluid^20^, and that impaired lung function is associated with obesity^21^. Chronic obstructive pulmonary disease, diabetes, hypertension and other aging-associated diseases, are associated with impaired vasoregulation and subclinical polyneuropathy of the sural nerve, radial nerve, and median nerve^22^,^23^,^24^, nearby BL 56–57, LU 6–7, and PC 5–7, respectively. International studies have shown that phenomena of meridians exist in both humans and animals^1^,^5^,^6^,^7^. Recent studies have shown a similarity between the meridian system and the primo-vascular system, a novel vascular network found in and on most organs as well as inside certain lymph and blood vessels^25^. However, the connection and function between the somatic meridians and internal organs, especially the structure and biomolecules of the pathway, still remain unclear. A more sophisticated approach including measurement of the corresponding organs’ functions would be required to address this issue. Despite these limitations, our findings suggest a non-systemic NO bioavailability over specific skin region of LU meridian associated with aging, which may involve potential pathophysiological changes in the lung.

Our previous studies reported that NO levels are elevated over PC and BL regions in overweight/obese subjects, and conversely, cGMP levels are decreased over the ventral forearm PC acupoints in overweight/obese subjects^11^. The present results show that NO content over the BL meridian is elevated in older overweight/obese subjects compared to younger subjects. However, there are no significant differences of NO levels over PC regions in elder compared to younger subjects in normal weight and overweight/obese groups, respectively. The findings are consistent with our previous report that NO is enhanced over PC and BL regions in overweight/obesity, and further suggest that enhanced NO levels predominantly exist over BL region in older overweight/obese subjects. Catecholamine receptor desensitization is associated with aging, and age is related to bladder dysfunctions such as incontinence, a decrease in maximum urethral closing pressure, apoptotic loss of striated muscle fibers, large residual urine volumes after urination, and detrusor hyposystole^26^. In addition, obesity is associated with higher risk of urinary incontinence in both males and females in adults^27^,^28^. NO activates cGMP, the main intracellular second messenger that produces relaxation of urethral smooth muscle and is involved in regulation of detrusor tone^29^. The present data supports a non-systemic association in NO bioavailability over specific skin regions and increased NO release over BL regions with aging, which is potentiated by overweight/obesity. Whether selectively increased NO levels along the BL meridian in elder subjects reflect a high NO level in somatic-bladder organ to counteract the dysfunction process or sympathetic activity desensitization with aging requires further investigation.

Moreover, the present results show that NO_x_^−^ concentrations are higher over the PC region in overweight/obese males compared to females, but are not different in race over both PC and BL regions. However, there are no detectable differences of NO levels over PC regions between adults vs. elder subjects in normal weight subgroups and overweight/obese subgroups. The results suggest that enhanced NO over PC regions in overweight/obese subjects is male-gender dependent, which are not influenced by elder-age. The results also demonstrate that NO levels over PC regions between normal weight and overweight/obese subjects are not altered in adult females groups but were marginally increased in adult males of overweight/obese subjects. Systolic and diastolic arterial blood pressure is also higher in overweight/obese subjects than those in normal weight subjects. Consistently, the PC meridian is associated with the heart and acupoint PC 6 is commonly used to treat cardiovascular diseases including hypertension and coronary artery disease in TCM^2^. Our chemical assay results are consistent with the data from physiological record of arterial blood pressure and further suggest that overweight/obesity is an important factor for modification of NO level over PC meridian region. Increasing clinical reports over the past several decades reveal a higher mortality and cardiovascular events in female vs. male patients with ischemic heart diseases^30^,^31^,^32^. The underlying mechanisms responsible for the difference remains unknown, although variation in risk factors, sex hormones, and different pathophysiological changes including more severely impaired NO-dependent coronary vasodilation in females vs. males have been proposed^31^,^32^. The findings presented suggest that enhanced NO over PC regions in overweight/obese males may be involved in potential pathophysiological changes in PC meridian-heart interaction, which provide protective influence and contribute to the difference between females and males. Males have significantly higher sweat rates on the forearm, forehead, chest, and back as compared to females^33^,^34^. In both genders, the skin, a steroidogenic tissue, metabolizes and responds differently to sex hormones. Males have thicker and darker skin, and also differ in testosterone influenced hair growth, increased sebum production and pore size, higher surface pH, less subcutaneous fat accumulation, and less serum leptins^34^. Recent studies demonstrate a link between excessive sweating or hyperhidrosis in obesity^35^. Several reports have demonstrated that NO can be produced non-enzymatically through reduction of nitrate by bacteria, which may colonize in skin areas rich in hair follicles and sweat glands^12^,^13^,^14^. Our previous studies showed that nonenzymatic reduction of nitrate by bacteria plays an important role in generation of NO on skin acupoint/meridians^17^,^18^. The present results suggest that higher sweat rates on the forearm in overweight/obese males^33^,^34^,^35^ cause more nonenzymatic production, which plays a major role in enhanced NO levels over the PC region located on the medial side of the ventral forearm, in addition to differences in skin thickness and hormones between male and female.

The effect of genetic and environmental factors, including pathology, on the bioavailability of NO and other metabolites on the skin is not well characterized. The skin is a unique non-systemic site for analysis of biomolecules. Cutaneous measurement of sweat and sebaceous proteins and other biomolecules has recently been explored in studies as potential pathological indicators^36^,^37^. Diseases can alter sweat composition through various mechanisms, and thus the proteomic and metabolomic profile of eccrine sweat may serve as a potential biomarker of specific diseases^36^,^37^. Despite the unique properties of eccrine sweat and its potential association with the physiological state of diverse tissues, there are unfortunately relatively few studies that characterize NO and metabolites over skin^36^,^37^,^38^,^39^. The present results demonstrate the alterations of topographical distribution of NO levels over specific somatic skin regions of the meridians, which may be involved in their potential pathophysiological changes in their corresponding organs with aging and obesity. Previous studies have shown that acupoints and meridian lines possess characteristics of low electrical resistance and high electric currents in both humans and animals^1^,^40^,^41^. Our recent studies have demonstrated that L-arginine-derived NO synthesis increases skin electric currents over acupoints in rats^42^. These findings suggest that NO may mediate connections between visceral organs and somatic meridians associated with the pathophysiological alterations of the meridian system described in TCM. In addition, our results show that the alternations of NO levels with elder subjects and males of overweight/obese subjects exist along a region of the meridian pathway including the non-meridian area adjacent to the meridian. Previous studies reported that the width of the propagated sensation along channels over the body surface of humans occurred about 0.5 to 5.0 cm, and the width of a number of skin diseases along meridian pathways existed about 0.5 to 2 cm^43^, which are defined to be consistent with the course of the classical meridians^7^,^43^. The width of non-meridian area defined in our studies is about 1 to 1.5 cm adjacent to the meridian line, which is within the regions of meridian lines reported. Our results agree with previous studies reporting that width of the meridian is a non-defined region^43^, and further suggest that the regions involved in alterations of NO levels over LU and PC meridians become wider to cover the adjacent non-meridian area in pathological conditions than those at physiological status; however, such changes are still distributed over enlarged meridian areas. Whether pathological conditions cause an enlarged distribution of NO and related biomolecules along meridian pathways and whether biochemical mechanisms of NO are responsible for somatic-organ connections/alterations in the meridian system which contribute to visceral organ function/disease, is another important objective.

In summary, our findings from the biocapture analysis show that NO levels over the LU meridian region are selectively decreased in older subjects. In overweight/obese subjects, NO contents are increased over the BL meridian region in older subjects and over the PC meridian region in males compared to females. In conclusion, these results suggest that the topographical distribution of NO on the skin regions is associated with aging, gender, and obesity. Though the underlying evidence from measurements of corresponding organs’ functions to support the NO bioavailability over the selected meridians reflecting somatic-organ connections are not fully illustrated in the current study, the findings of this study will shed light on selective association between aging and NO bioavailability over LU and BL meridian regions as well as specific influence of gender on NO distribution over PC meridian regions that may support somatic-organ connections and reflect aging/gender-associated organ-specific alterations.

## Methods

### Human Subjects

Sixty one men and women (18- to 75-years-old) recruited at Harbor-UCLA Medical Center volunteered for the following studies. All participants were healthy nonsmokers who did not have major surgery in the past 12 months nor history of cardiovascular disease. Subjects with dermatological problems, allergic diseases, vascular disorders, infectious diseases, and prescribed medication were excluded from the study. The protocol was approved by the John F. Wolf, MD Human Subjects Committee of the Los Angeles Biomedical Research Institute at Harbor-UCLA Medical Center, and all experiments were performed in accordance with relevant guidelines and regulations. Subjects were given a detailed oral instruction of the study, and informed consent was obtained from all subjects. Female participants were not on their menstrual period on the day of study. Experiments were performed in a quiet, air-conditioned room with the temperature maintained at 25–27 °C. All subjects were instructed to maintain their regular diet of breakfast or lunch, but to not eat or drink 2 hours prior to the test.

### Identification of Acupoints, Instrumentation, and Biocapture over Skin

Acupoints, MWOP, and NMCR over PC, BL, and LU meridians were studied in subjects as described in Fig. 1. Locations were identified by an acupoint/meridian map of the human body^2^. Regions were chosen based on consistency in identification and sufficient spacing for NO biocapture tube placement without contacting other meridians^11^,^18^.

The biocapture method was described previously in humans^11^,^18^. Sterile distilled water was swabbed onto the skin surface along the PC, BL, and LU meridian and NMCR 2 times at 5 min intervals. A biocapture device, developed by this lab, consists of a semi-circular molded plastic tube (0.5 × 5 cm) adhered to the acupoints, MWOP, and NMCR by a custom double-sided adhesive, as shown in the top central and bottom left panels of Fig. 1. As shown in Fig. 1, biocapture tubes are adhered to the skin surface over acupoints (PC 5–7, LU 8–9, or BL 57), MWOP (defined as the area between PC 3–4, LU 6–7, or BL 55–56), and adjacent to the defined acupoints as the non-meridian control. PTIO solution (100 μM 2-Phenyl-4,4,5,5-tetramethylimidazoline-1-oxyl 3-oxide, purchased from Sigma-Aldrich) was injected inside the sterilized tube to absorb NO from the skin surface for 20 minutes. After incubation, the liquid was collected from the tubes. Biocaptured samples were transferred to plastic vials and stored in −80 °C, for measurements of total nitrite plus nitrate (NO_x_^−^) concentrations. The concentrations of NO_x_^−^ in collected samples were then quantified in a blinded fashion^11^,^17^,^18^.

### Quantification of NO Metabolites

The total NO_x_^−^ concentration was measured in the biocapture solution using an ozone phase chemiluminescence method (NOA280i, GE Analytical Instruments, Boulder, CO) as described previously^11^,^17^,^18^. Samples containing NO_2_^−^ and NO_3_^−^ were reduced to NO gas, which can be quantified by the chemiluminescence detection device after reaction with ozone. Briefly, NO_x_^−^ concentrations were measured by using refluxed 1.5 mM vanadium (III) chloride in 2 M HCl at >90 °C with a circulating water bath and cold water for the condenser, which quantitatively reduces both NO_2_^−^ and NO_3_^−^ to NO gas. Refluxing 1% potassium iodide in glacial acetic acid causes a rapid one-electron reduction of NO_2_^−^ to NO gas and this acidification/reduction solution was used to determine NO_2_^−^ concentrations. Values for NO_2_^−^ and NO_x_^−^ were quantified, and values for NO_3_^−^ were calculated by subtracting NO_2_^−^ from NO_x_^−^ values. The nitrate and nitrite calibration curves were established using known concentrations of NO_3_^−^ (NaNO_3_) and NO_2_^−^ (NaNO_2_), respectively. The linearity of the standard curves was made each day, and the quantitative analyses were based on measurements of peak areas of the standard compounds. All samples were measured in duplicate and conducted in a blinded manner. The presence of (NO_2_^−^) in our samples was close to the water basal level. Therefore, the final NO_x_^−^ concentration in the solution was expressed as μM with no allowance made for NO_2_^−^. The lower limit of the detection of this assay was 0.1 pmol of NO.

### Research Protocols

Volunteers were randomly asked to participate in biocapture over the PC, BL, or LU meridian region. BMI was calculated by dividing the weight (in kg) by the squared height (in meters). BMI >25 is considered as overweight and over 30 as obese. Acupoints on skin surface of PC 5–7, BL 57, and LU 8–9, and their MWOP and NMCR were determined following the procedure described above^2^,^11^,^18^. The biocapture device was attached to the skin surface over the PC, BL, and LU regions containing acupoint, MWOP, and NMCR, as shown in Fig. 1. PTIO solution (100 μmol/l) was placed inside the tubing and attached to the surface of the skin for 20 min. The liquid was drained from the tubing and kept in −80 °C. The concentrations of NO_x_^−^ in the samples were quantified by using a chemiluminescence NO analyzer. The assays were conducted in a blinded fashion.

### Statistical Analysis

Results were expressed as mean ± standard error of the mean (SEM) of NO_x_^−^ concentrations over the skin surface measured in the biocapture solution (μM) and efflux rate calculated over the surface area during 20 min (nmol/cm^2^) along the skin surface in contact with the solution, respectively. The significance of differences was determined by two factor-repeated Analysis of Variance (ANOVA), where the two factors are (1) age, gender or race; and (2) three sites: acupoints, MWOP, NMCR over the PC, BL or LU meridians. P values less than 0.05 were considered significant.

## Additional Information

**How to cite this article**: Ma, S.-X. *et al.* Influence of age, gender, and race on nitric oxide release over acupuncture points-meridians. *Sci. Rep.* **5**, 17547; doi: 10.1038/srep17547 (2015).

## Figures and Tables

**Table 1:** Characteristics of participants.

Characteristics	Normal Weight (n = 30)	Overweight/Obesity (n = 31)
Women, No.	22	16
Men, No.	8	15
Age, mean (SEM), [<40 years]	24.5 ± 1.4	28.8 ± 1.2
Age, mean (SEM), [≥40 years]	54.3 ± 3.2	51.4 ± 2.5
Body Mass Index, mean (SEM)	21.3 ± 0.4	30.0 ± 0.9^*^
Systolic BP (mm Hg), [<40 years]	108.6 ± 2.6	121.0 ± 2.1^*^
Systolic BP (mm Hg), [≥40 years]	125.9 ± 7.0^#^	124.7 ± 6.9
Diastolic BP (mm Hg), [<40 years]	60.6 ± 1.2	66.6 ± 1.0^*^
Diastolic BP (mm Hg), [≥40 years]	70.6 ± 4.9	75.9 ± 4.8
White	7	15
Asian	19	9
Black or African American	3	3
Pacific Islander	1	2
Native American	0	1
More than One Race	0	1
Hispanic	24	26
Non-Hispanic	6	5

*P < 0.05, compared to normal weight subjects; #P < 0.05, compared to subjects < 40 years.
